# Why I decide to leave South Korea healthcare system

**DOI:** 10.1016/j.lanwpc.2024.101232

**Published:** 2024-10-30

**Authors:** Jounggi Moon, Joo-Young Lee

**Affiliations:** a Withus Nursing Hospital, Gyeongju-si, Gyeongsangbuk-do, Republic of Korea; b Department of Psychiatry, University of Colorado School of Medicine, Aurora, CO, USA

I (JM) was a neurosurgery resident in South Korea until February 2024, when the government announced a plan to increase the medical student quota by 66%, immediately in 2025. In response, over 90% of resident physicians resigned^1^; I was among them. This is my personal story—how the new healthcare policy compelled me to relinquish my pursuit.

I began my residency training in 2023, drawn to the role of a brain surgeon: performing surgery on critically ill patients, providing intensive post-operative care, and saving lives. Despite the incredible challenges of neurosurgery residency, I found fulfillment in the gratitude expressed by patients and their families for the lives I helped save.

The government anticipated that this expansion would saturate preferred specialties, forcing the remaining physicians to “trickle down” into less desirable fields of essential medicine.^2^ However, the unprecedented rate of medical school expansion was not supported by any scientific projections.

I resigned in protest of the government's unsubstantiated and one-sided healthcare reform. My medical license could not be affiliated with two medical institutions simultaneously by the law. The executive orders forced my license to be bound to my previous training institution, disabling other hospitals to hire me as a generalist. The government threatened to suspend my hard-earned medical license.^2^ After five months in limbo, my resignation request was officially processed in July 2024. I am now employed at a non-teaching hospital.

Meanwhile, major media outlets portrayed the resigned residents as criminals and traitors, accusing us of “abandoning patients for monetary gain.” The demonization of physicians was intense. Did my resignation deserve such treatment? Am I being condemned as selfish for—out of hopelessness—refusing to work 80-h weeks at below-minimum hourly rates?

My world has been upended. I worry that my patients will remember me as someone who left them for money. Would those I cared for during residency see me as an opportunist? Was it foolish to believe that I had genuinely helped them? I fear I may never connect sincerely with patients again. I feel broken. I cannot see myself as a practicing physician in Korea.

South Korea's universal healthcare system faces sustainability challenges. It has overly relied on the labor of trainees to cut costs. We resigned when the reform suggested an even heavier dependence on this strategy. With the nation's fertility rate at the lowest in the world^3^—leading to fewer potential payers—the health insurance fund is on the brink of insolvency, likely heading into the red within five years.^4^ It is crucial for government officials, physicians, medical students, and the public to engage in extensive discussions about the future direction of the country's healthcare. However, given the trajectory of reform, I have lost hope that physicians' voices will be heard in policymaking.

The announcement of the reform has prompted many Korean physicians to consider practicing abroad. After much contemplation, I have decided to give up my physician role within the Korean healthcare system and began preparing for relocation abroad, seeking a safer, more respected, and predictable clinical environment.

## Contributors

Dr. Jounggi Moon conceptualized and wrote the first draft of the manuscript, incorporating personal experiences and medical expertise on South Korea's healthcare system. Dr. Joo-Young Lee provided critical revisions, particularly in structuring the narrative and enhancing readability. Both authors reviewed and approved the final version of the manuscript.

## Declaration of interests

Dr. Jounggi Moon was previously a neurosurgery resident at The Catholic University of Korea Uijeongbu St. Mary's Hospital, resigning in February 2024. The resignation was processed officially in July 2024. Dr. Jounggi Moon has been employed as a doctor on duty at Withus Nursing Hospital since August 2024.

We declare no competing interests.
